# The association between cognitive frailty and the risk of fall occurrence in older adults: a meta-analysis of cohort studies

**DOI:** 10.3389/fmed.2025.1537240

**Published:** 2025-02-12

**Authors:** Jian Liu, Yu Wu, Zongke Long, Simeng Zhang, Shicai Wu

**Affiliations:** ^1^School of Nursing and Rehabilitation, Cheeloo College of Medicine, Shandong University, Jinan, Shandong, China; ^2^University of Health and Rehabilitation Sciences, Qingdao, Shandong, China; ^3^China Rehabilitation Research Center, Beijing Bo’ai Hospital, Beijing, China

**Keywords:** cognitive frailty, falls, meta-analysis, older adults, systematic review

## Abstract

**Background:**

Cognitive frailty increases the risk of fall occurrence. However, previous studies have shown inconsistent correlations between cognitive frailty and the risk of fall occurrence.

**Objective:**

To systematically review studies and explore the association between cognitive frailty and the risk of fall occurrence.

**Methods:**

Databases were systematically searched. Meta-analysis was performed using RevMan 5.4 software after evaluation of the quality of the included studies by 2 researchers.

**Results:**

A total of five studies including 16,962 patients were included. The results of Meta-analysis showed that the cognitive frailty group increased the risk of occurrence of falls in older adults [OR = 1.38, 95% CI (1.09, 1.73), *p* = 0.006]. Subgroup analyses showed that cognitive frailty in older adults increased the risk of fall occurrence using different cognitive frailty assessment tools, study participants from the community, different regions, and different sample sizes.

**Conclusion:**

The results of this study suggest that cognitive frailty in older adults is an independent risk factor for the occurrence of falls, and it is recommended that caregivers strengthen the assessment of cognitive aspects of older adults admitted to the hospital.

## Highlights

- In this systematic review and meta-analysis, we considered for the first time the possible relationship between cognitive frailty and the occurrence of falls in a cohort study. The results of this study suggest that cognitive frailty in older adults is an independent risk factor for the occurrence of falls.
- It is recommended that caregivers strengthen the assessment of cognitive aspects of older adults admitted to the hospital so that they can accurately identify those who are at high risk for falls.
- Different CF assessment tools were used in the included studies and there was a certain recall bias in the assessment of falls, which had an impact on the result.

## Introduction

A fall is usually defined as an event in which an individual falls to the ground or other lower plane as a result of an unintended fall (1). The incidence of falls increases with age, and preventing and managing falls has become a challenge for society. According to the World Health Organization, the percentage of older adults who have experienced at least one fall in a year is 30% (2). Falls in older adults can lead to fractures, joint dislocations and disability, which can lead to increased dependency and decreased quality of life and even death (3). Early identification of fall-related risks in older adults and intervention is crucial for caregivers. In 2013, the International Association of Gerontology first proposed the new concept of Cognitive Frailty (CF), which refers to the combination of frailty and mild cognitive impairment (MCI) in the exclusion of neurodegenerative disorders such as dementia (4). A Meta-analysis showed that frailty was significantly associated with a high risk of falls occurring (5). Another Meta-analysis of 27 studies found that community-dwelling older adults with MCI had more than twice the risk of fall occurrence than cognitively normal older adults (6). The introduction of the concept of cognitive frailty combines the two, emphasizing the co-occurrence of frailty and MCI, which, at a theoretical level, would significantly increase the occurrence of falls in older adults. A Meta-analysis showed that CF not only increases the risk of dementia, but also increases the risk of adverse outcomes such as death (7). However, in various current studies, the findings are not entirely consistent (8–10).

In current clinical care, falls assessment has become a routine item in the admission assessment. The Morse Falls Risk Assessment Scale is the most commonly used in clinical practice and is currently considered the best predictive tool. The scale has six entries, but four of the scale’s entries focus on frailty aspects of the older adult’s characteristics, less on cognitive aspects, and only one question in the last entry asks about good mental status. In addition, assessments of cognition are rare in current admission routine nursing assessments. As mentioned earlier, the impact of MCI on falls has become prominent and there is a need to strengthen the assessment of cognition in the nursing assessment of older adults on admission. Therefore, this study will systematically evaluate the relationship between CF and the risk of fall occurrence in older adults with the aim of determining whether CF is an independent risk factor for falls, thereby drawing attention to the cognitive aspect of clinical care. The assessment of CF may provide intervenable targets for clinical caregivers to reduce the risk of fall occurrence in older adults.

## Methods

### Design

A systematic review and meta-analysis were conducted according to the Preferred Reporting Items for Systematic Review and Meta-Analysis (PRISMA) statements.

### Search strategy

**Figure 1:**
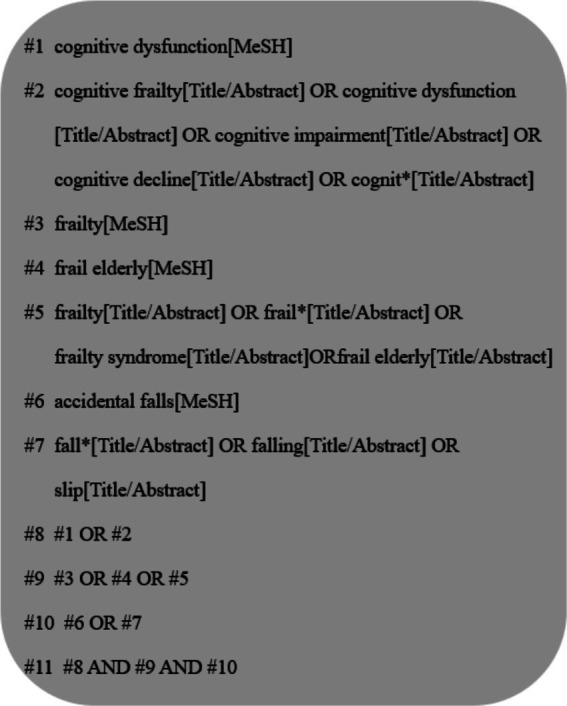
PubMed search strategy.

The system searched Cochrane Library, PubMed, Embase, Medline, CINAHL, Scopus, Proquest Central, Web of Science, SinoMed, CNKI, VIP and Wan fang Databases. The search period was from the establishment of the database to December 2023, and databases were searched by “cognitive frailty/cognitive dysfunction/cognitive impairment/cognitive decline/cognit^*^” “frailty/frail^*^/frailty syndrome/frail elderly” “accidental falls/fall^*^/falling/slip” as search terms. Using PubMed as an example, its specific search strategy is shown in Figure 1.

### Eligibility criteria

Literature Inclusion Criteria: (1) Study population: older adults aged ≥60 years; (2) Exposure factors: CF was assessed using the 2013 International Consensus Panel (4), which proposes the co-existence of frailty and MCI in the exclusion of neurodegenerative diseases such as dementia. Among them, frailty was assessed using the current frailty assessment tools for the older adults that are used more at home and abroad, such as the Fried Frailty Phenotype Scale and the FRAIL Frailty Scale, etc. MCI was assessed using commonly used cognitive assessment scales, such as the Mini-mental State Examination (MMSE), cognitive tests or self-reports, Montreal Cognitive Assessment (MoCA), etc.; (3) Outcome indicator: occurrence of falls; (4) Study type: prospective cohort study on the relationship between CF and the risk of falls occurrence in older adults.

Literature exclusion criteria: (1) Data in the original study could not be converted and applied; (2) Data could not be extracted and duplicated literature.

### Literature screening and data extraction

Data from the selected studies were extracted from Excel spreadsheets by 2 researchers each. Any disagreements were resolved by consensus with the 3rd researcher. Basic information was extracted and cross-checked for the included literature, which included: first author and year of publication, region of investigation, source of study population, sample size, duration of follow-up, age, survey instrument for CF, fall survey instrument, OR and 95% CI, and adjustment factors.

### Literature quality evaluation

For accuracy, 2 researchers independently completed the data extraction process. Any data discrepancies could be resolved by referring to the original articles. Literature quality was evaluated using the Newcastle-Ottawa Quality Assessment Scale (NOS) (11), which includes three dimensions: study population selection, comparability, and outcome measures, with a total of 8 entries and a total score of 9. Articles with a score of 6 or higher were of high quality.

### Statistical analysis

This study used EndNote X9 software for literature management, extracted data using Excel sheets, and applied RevMan 5.4 software for Meta-analysis. The ending index was the occurrence of falls, and the combined effect size was expressed as the ratio (OR) and 95% CI, and the combined data were tested for heterogeneity and combined with *I*^2^ to evaluate the magnitude of heterogeneity. If *p* > 0.10 and *I*^2^ ≤ 50%, there was homogeneity among studies, and a fixed-effects model was selected for Meta-analysis; if *p* ≤ 0.10 and *I*^2^ > 50%, there was heterogeneity among studies, and a random-effects model was selected for Meta-analysis. Subgroup analysis was performed according to the basic characteristics of the included studies to explore and reduce heterogeneity, and sensitivity analysis was used to evaluate the stability of the results. Differences were considered statistically significant at *p* < 0.05.

## Results

### Study characteristics

**Figure 2:**
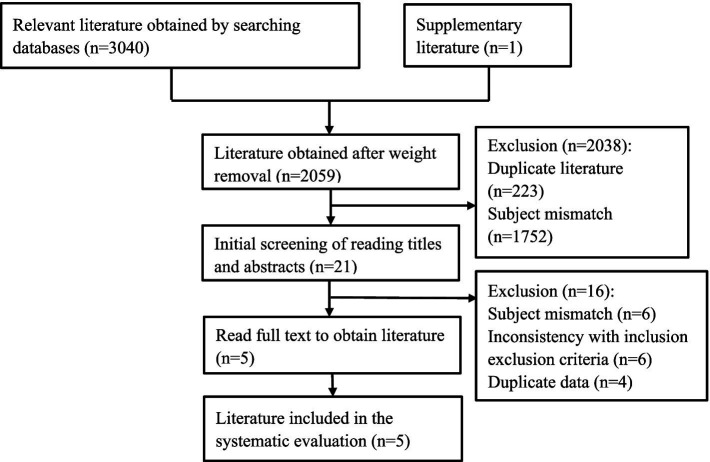
PRISMA flowchart.

According to the established search plan, a total of 3,041 studies (2,911 in English and 130 in Chinese) were obtained from the initial examination, and after screening according to the criteria, five (8, 9, 12–14) studies were finally included, all of which were in English. The literature screening process is shown in Figure 2.

### Basic characteristics of the included studies

Five (8, 9, 12–14) studies were included as prospective cohort studies with a total of 16,962 patients. 2 (9, 14) studies used the Fried Frailty Phenotypic Scale and the MMSE Scale as the assessment tools for CF; 1 (8) study used the Fried Frailty Phenotypic Scale and the Hasegawa Dementia Scale to assess CF; 1 (12) study used the FRAIL Frailty Scale and the Mini-Cog Scale to assess CF; and 1 (13) study assessed CF using the Fried Frailty Phenotype Scale and cognitive tests or self/proxy reports. Falls were assessed using a single entry asking about the occurrence of falls, which ranged from 10.6 to 47.6% (see Table 1).

**Table 1:** Basic characteristics of included studies.

First author and year of publication	Area of investigation	Source of study population	Sample size	Duration of follow-up	Age	Cognitive frailty instrument		Falls instrument	OR (95% CI)	Adjustment factors
						Physical frailty	Mild cognitive impairment			
Ma (8), 2021	China	Community	965	3 years	74.9 ± 3.7	Fried Frailty Phenotype Scale	Hasegawa Dementia Scale	Single-entry issues	3.11 (1.06, 9.15)	Gender, age, education level, occupation, marital status, BMI, smoking and alcohol history, health status, depression, diabetes, hypertension
Zhang (12), 2021	China	hospital	9,192	30 days	72.4 ± 5.7	FRAIL Frailty Scale	Mini-Cog Scale	Single-entry issues	3.00 (1.32, 6.83)	Age, gender, education level, depression, ward aggregation effects
Ge (13), 2021	United States of America	hospital	6,000	6 years	≥65	Fried Frailty Phenotype Scale	Cognitive testing or self/proxy reports	Single-entry issues	1.28 (1.17, 1.40)	Age, gender, race, education level, living alone, obesity, co-morbidities, mobility impairments
Rivan (14), 2021	Malaysia	Community	400	5 years	69.0 ± 6.2	Fried Frailty Phenotype Scale	MMSE Scale	Single-entry issues	2.98 (1.78, 4.99)	Age, gender, education level, waist circumference, loneliness status
Brigola (9), 2020	Brazilian	Community	405	4 years	70.6 ± 7.1	Fried Frailty Phenotype Scale	MMSE Scale	Single-entry issues	1.44 (0.51, 4.05)	Gender, age, education level, BMI

### Evaluation of the quality of included studies

The included studies were evaluated for quality on the NOS scale and scored 7–9, indicating that the included studies were all high-quality literature, with one study (8) scoring a perfect score (see Table 2).

**Table 2:** Quality assessment of included studies.

Inclusion in the study	Selection of study participants				Comparability between groups	Outcome measures total score			Score
	Representativeness of the exposed group	Representativeness of the non-exposed group	Exposure factors determined	Exposure factors determined no outcome measures to observe at the time of the study		Whether outcome evaluations were independent	Whether follow-up was long enough	Follow-up completeness	
Ma (8)	1	1	1	1	2	1	1	1	9
Zhang (12)	1	1	1	0	2	1	0	1	7
Ge (13)	1	1	1	0	2	1	1	1	8
Rivan (14)	1	1	1	0	2	1	1	0	7
Brigola (9)	1	1	1	0	2	1	1	0	7

### Relationship between CF and the occurrence of falls

**Figure 3:**
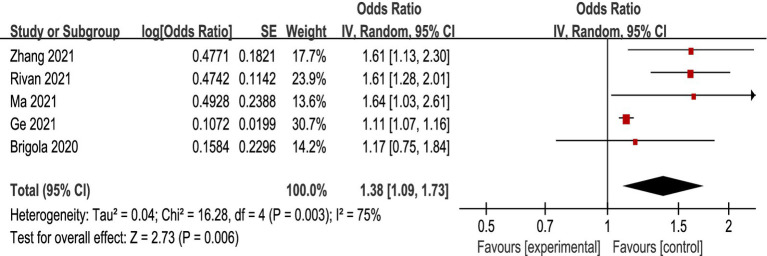
Association between CF and the occurrence of falls.

There was heterogeneity among the included studies (*p* = 0.003, *I*^2^=75%), so a random effects model was chosen for Meta-analysis. The results showed that the CF group increased the risk of fall occurrence compared to the non-CF group [OR = 1.38, 95% CI (1.09, 1.73), *p* = 0.006], and the difference was statistically significant (see Figure 3).

### Subgroup analysis

In order to further identify the reasons for heterogeneity, this study explored the relationship between CF and the risk of fall occurrence in older adults in 4 aspects (CF assessment tool, source of study participants, region, and sample size) based on the basic characteristics of the included studies. (1) CF assessment tools: 2 studies (9, 14) CF was assessed using the Fried Frailty Phenotype Scale and the MMSE Scale, and the rest of the studies used the more commonly used CF assessment tools. Meta-analysis showed that the use of the Fried Frailty Phenotype Scale and the MMSE Scale, as well as the other combinations of the assessment tools, indicated that older adults with CF were at increased risk of falls. (2) Source of study population: three (8, 9, 14) studies were community-sourced and two (12, 13) were hospital-sourced. Meta-analysis showed that hospital-sourced CF in older adults did not increase the risk of falls [OR = 1.28, 95% CI (0.90, 1.82), *p* = 0.17], and community-sourced CF in older adults was associated with an increased risk of falls. Associated with an increased risk of falls. (3) Region: 3 studies (8, 12, 14) were in Asia, and 2 studies (9, 13) were in the Americas. Meta-analysis of the results showed that CF in older adults in different regions increased the risk of fall occurrence. (4) Sample size: 2 studies (9, 14) had a sample size of less than 900, and 3 studies (8, 12, 13) had a sample size of more than 900. Meta-analysis of the results showed that sample size did not affect the correlation between CF in older adults and an increased risk of fall occurrence (see Table 3).

**Table 3:** Subgroup analyses of the association between CF and the risk of fall occurrence.

Criteria for grouping	Number of studies included	Confluence effect size		Heterogeneity test	
		*p*	OR (95% CI)	*p*	*I*^2^ (%)
Cognitive frailty assessment tool					
Fried Frailty Phenotype Scale + MMSE Scale	2	<0.001	1.71 (1.24, 1.84)	0.22	34
Fried Frailty Phenotype Scale + Cognitive performance tests or self/proxy reports	1	<0.001	1.11 (1.07, 1.16)	-	-
Fried Frailty Phenotype Scale + Hasegawa Dementia Scale	1	0.04	1.64 (1.03, 2.61)	-	
FRAIL Frailty Scale + Mini-Cog Scale	1	0.009	1.61 (1.13, 2.30)	-	-
Source of research subjects					
Hospitals	2	0.17	1.28 (0.90, 1.82)	0.04	75
Community	3	<0.001	1.53 (1.27, 1.84)	0.45	0
Region					
Asia	3	<0.001	1.61 (1.35, 1.92)	1.00	0
Americas	2	<0.001	1.11 (1.07, 1.16)	0.82	0
Sample size					
<900	2	<0.001	1.71 (1.24, 1.84)	0.22	34
≥900	3	<0.001	1.35 (1.00, 1.83)	0.04	70

### Sensitivity analysis and publication bias

Sensitivity analyses were performed after excluding each of the five prospective cohort studies whose outcome indicator was the occurrence of falls, and the results showed no significant change in the amount of the combined effect, indicating that the results of the Meta-analysis of this study were essentially stable. Publication bias was not analyzed because the number of included studies was small and did not meet the minimum number of documents for making a funnel plot.

## Discussion

A total of 5 prospective cohort studies were finally included in this study, and the scores of the included literature were rated from 7 to 9 after standardized evaluation, of which, 2 studies (8, 13) scored 8–9, suggesting that the overall quality of the included literature was high. The included studies all used the more commonly used current CF and falls assessment tools, and each study controlled for the influence of confounding factors on the results, and the results also showed that the results of this study were relatively stable after the exclusion of each study. Based on this, the results of the Meta-analysis of this study have high reliability. However, because all of the fall assessments in this study were single-entry inquiries about falls and relied on patient self-reporting, there was some recall bias and the effect of the remaining confounders, and the results of this study need to be viewed with caution.

A cross-sectional study (10) showed that CF was associated with falls in community-dwelling Japanese older adults. However, Brigola et al. (9) did not find an association between CF and falls after a 4-year follow-up of 405 older adults. The results of this study showed that patients in the CF group had a 1.38-fold increased risk of falls compared with patients in the non-CF group, suggesting that CF is a risk factor for the occurrence of falls in older adults.

The mechanisms underlying the association between CF and falls in older adults are complex: slow gait speed, a key feature of frailty, is associated with cognitive deficits in processing speed, attention, and executive function (15), and cognitive deficits and resulting declines in cognitive reserve may make it difficult to ameliorate frailty (16); another study (17) found that both frailty and MCI are associated with higher levels of inflammatory markers, and that CF significantly elevated inflammatory markers in older adults may trigger the onset of falls by accelerating muscle loss and impairing muscle tissue regeneration; and research (18) suggests that older adults with CF often experience reduced nutrients or visual impairment, and are less responsive when presented with a new environment, making them susceptible to falls. These results suggest that geriatric syndromes such as frailty and MCI may represent common pathways that modulate the association between individual risk factors and falls, presenting a synergistic effect on falls. Therefore, when older adults have these two geriatric syndromes, they are more susceptible to falls, early screening for CF may help to prevent falls, and the assessment and management of CF should be emphasized among clinical caregivers.

Subgroup analyses in this study showed that older adults assessed as having CF using the Fried Frailty Phenotype Scale and the MMSE Scale assessment tools were at higher risk for falls; that community-dwelling older adults with CF had a significantly increased risk for falls compared to those in hospital settings; and that neither the size of the sample from different regions nor the size of the sample from different studies affected the relevance of the increased risk for falls in older adults with CF.

The Fried Frailty Phenotype Scale was proposed by Fried et al. (19) in 2001 based on the theory of the cycle of frailty, including five assessment indicators such as unconscious weight loss, and the Asia-Pacific Clinical Practice Guidelines for the Management of Frailty in the Older stated that the use of this scale is effective in predicting the risk of falls in older adults, and it is one of the internationally recognized tools for the assessment of frailty. The MMSE Scale was developed by Folstein et al. (20) in 1975, and several studies have used this combined scale for CF assessment. Kim et al. (21) screened 1,248 community-dwelling older adults using this combined scale and found a correlation between CF and falls. The results of the subgroup analysis in this study found that community-dwelling older adults with CF had a significantly increased risk of falls compared to those in the hospital setting, possibly due to the higher prevalence of CF among older adults in the community (7), which, combined with the greater range of motion of older adults in the community setting, puts them at increased risk of falls.

The present study has different findings from previous studies after subgroup analysis of the source of the study population. The results of this study showed a nonsignificant relationship of increased risk of fall occurrence in older adults with CF in the hospital setting, which we consider to be a meaningful finding due to the paucity of current research on this outcome. It is hypothesized that the possible reasons for this are that in the hospital setting, clinical staff are more timely in assessing and teaching falls; secondly, falls are recognized as a major event affecting the safety of older adults admitted to the hospital, and nurses, as the main promoters of patient safety, will enhance care for falls; finally, older adults admitted to the hospital are usually in relatively serious condition, and they may have a limited ability to get out of bed, which may also reduce the incidence of falls to some extent. In other words, this also proves that CF is very significantly related to the occurrence of falls, and that CF is a strong predictor of falls, and timely intervention can reduce the incidence of fall events. Both Zhang (12) and Ge (13) et al. demonstrated that elderly patients with CF are at a higher risk of falling compared to those healthy hospitalized elderly patients, which suggests that screening for CF is important for the prevention of falls in hospitalized elderly patients. Therefore, the assessment of CF and management of falls in both community and hospitalized older adults should be strengthened.

### Implications of this study for clinical practice and future research

Based on the results of this study, CF in older adults significantly increases the risk of falls, and CF in older adults increases the risk of falls using different CF assessment tools, different sources of study participants (other than hospitals), different regions, and different sample sizes. This implies that clinical caregivers need to assess CF and implement appropriate interventions to enhance early warning awareness, prevention, and management when older adults are admitted to the hospital. There are fewer and limited quality studies related to CF interventions, and one study found that physical activity moderated the relationship between CF and falls (22), and activity participation can be considered as an effective way to improve CF. In addition, studies have shown that intervention programs including exercise training, nutrition programs, and memory training can reverse debilitation and cognitive impairment (13). Therefore, appropriate exercise training and nutritional interventions need to be considered in conjunction with inpatient fall-related education at discharge to reduce the incidence of falls.

### Limitations of this study

Only Chinese and English literature was included in this study, which may have language bias; fewer studies were included and the sample size for subgroup analysis was smaller; different CF assessment tools were used in the included studies and there was a certain recall bias in the assessment of falls, which had an impact on the results.

## Conclusion

The present study is based on a Meta-analysis of prospective cohort studies, and the results show that CF in older adults is an independent risk factor for the occurrence of falls. This suggests that nursing assessment of cognitive aspects should be strengthened at the time of hospital admission in older adults, and attention should be paid to the cumulative risk of cognitive emergence in debilitating situations, thus reducing the incidence of falls and improving the quality of care.

## Data Availability

Publicly available datasets were analyzed in this study. This data can be found here: the data that support the findings of this study are available from the corresponding author on reasonable request.
